# Exploring the views of healthcare professionals on increasing smoking cessation advice for patients

**DOI:** 10.1186/1753-6561-7-S1-P6

**Published:** 2013-01-30

**Authors:** ShuYing Ho, Hannah McGee, Noel G McElvaney, Frank Doyle

**Affiliations:** 1 Royal College of Surgeons in Ireland, Dublin, Ireland

## Background

Smoking cessation advice provided by healthcare professionals can be effective in increasing smoking cessation among patients. Any successful intervention will require staff knowledge of local barriers to implementation. However, the views of Irish healthcare professionals in increasing provision of smoking cessation advice and these barriers are unknown. The aim of this qualitative study is to explore the views of Irish healthcare professionals on barriers in increasing smoking cessation advice for patients in a large Irish teaching university hospital.

## Methods

Semi-structured interviews were conducted with 16 healthcare professionals recruited in Beaumont Hospital.

## Results

The main barriers identified are patient and staff attitude, time and service constraints, information not readily available and issues on smoke-free campus policy.

## Conclusions

Our results identified barriers expressed by Irish healthcare professionals in providing smoking cessation advice to patients. This supports the need to implement a multi-component hospital-based intervention to increase the rate of provision of smoking cessation advice in patients by healthcare professionals.

